# Functional–Structural Plant Models Mission in Advancing Crop Science: Opportunities and Prospects

**DOI:** 10.3389/fpls.2021.747142

**Published:** 2021-12-23

**Authors:** Soualihou Soualiou, Zhiwei Wang, Weiwei Sun, Philippe de Reffye, Brian Collins, Gaëtan Louarn, Youhong Song

**Affiliations:** ^1^School of Agronomy, Anhui Agricultural University, Hefei, China; ^2^The French Agricultural Research and International Cooperation Organization, Montpellier, France; ^3^College of Science and Engineering, James Cook University, Townsville, QLD, Australia; ^4^INRA, UR4 URP3F, BP6, Lusignan, France; ^5^Centre for Crop Science, The University of Queensland, Queensland Alliance for Agriculture and Food Innovation, Brisbane, QLD, Australia

**Keywords:** functional-structural plant modeling, plant architecture, plant phenotyping, genotype to phenotype, assisted molecular breeding

## Abstract

Functional–structural plant models (FSPMs) have been evolving for over 2 decades and their future development, to some extent, depends on the value of potential applications in crop science. To date, stabilizing crop production by identifying valuable traits for novel cultivars adapted to adverse environments is topical in crop science. Thus, this study will examine how FSPMs are able to address new challenges in crop science for sustainable crop production. FSPMs developed to simulate organogenesis, morphogenesis, and physiological activities under various environments and are amenable to downscale to the tissue, cellular, and molecular level or upscale to the whole plant and ecological level. In a modeling framework with independent and interactive modules, advanced algorithms provide morphophysiological details at various scales. FSPMs are shown to be able to: (i) provide crop ideotypes efficiently for optimizing the resource distribution and use for greater productivity and less disease risk, (ii) guide molecular design breeding via linking molecular basis to plant phenotypes as well as enrich crop models with an additional architectural dimension to assist breeding, and (iii) interact with plant phenotyping for molecular breeding in embracing three-dimensional (3D) architectural traits. This study illustrates that FSPMs have great prospects in speeding up precision breeding for specific environments due to the capacity for guiding and integrating ideotypes, phenotyping, molecular design, and linking molecular basis to target phenotypes. Consequently, the promising great applications of FSPMs in crop science will, in turn, accelerate their evolution and *vice versa*.

## Introduction

Global human population is growing rapidly and has been estimated to reach nearly 10 billion by 2050. However, overall crop production at current rate is insufficient for such great population (Ray et al., 2013). Undoubtfully, the growing population requires extra food supply as well as high food quality, which is in a conflict with shrinking availability of farmland due to industrialization and urbanization (Karki et al., 2013). Concomitantly, climate change is alarming and causing more droughts, heat shocks, and floods, which may further compromise crop productivity and grain quality (Altieri and Nicholls, 2017; Webber et al., 2018; Ababaei and Chenu, 2020). Accordingly, both the growing population and climate change constitute a roadblock in ensuring food security, which urges to increase crop productivity under likely harsher environments in a sustainable way.

To address such challenges, efforts in breeding have been attempted to develop novel high-yielding varieties under unfavorable environments along with improved agronomic managements (Henry and Nevo, 2014). However, the efforts have been hindered by the complex traits in controlling high yielding and quality under abiotic stresses (Hammer et al., 2005). Therefore, the adoption of new techniques and tools, e.g., plant/crop growth models in dissecting complex traits into secondary traits that can be related to specific morphophysiological pathways and genes, is important in tackling such challenges in crop production systems (Hammer et al., 2006; Tardieu and Tuberosa, 2010; Rebolledo et al., 2015). Such models based on concepts rooted in robust system biology and open frameworks that allow integrating knowledge of plant behaviors and research hypothesis (Yin and Struik, 2010; Hammer et al., 2016) and will be particularly useful for studying the interaction of genotypes and environments (G × E) precisely and decoding complex traits (Messina et al., 2015). For instance, crop/plant models are shown with a great capacity in realizing such aims (Hammer et al., 2006, 2010; Letort et al., 2008). Nevertheless, plant architectural or related traits, e.g., leaf/root three-dimensional (3D) characteristics are not often taken into account in crop models though leaf area and leaf area index that are key determinants in such models.

On the other hand, as for the architectural trait, it is one of key drives in functional–structural plant models (FSPMs). The concept and definition of FSPMs have been clearly described in many places (Vos et al., 2010; DeJong et al., 2011; Sievänen et al., 2014). Hence, in this study, we describe the model in brief only. FSPMs are dedicated in the simulation of both the plant architectural development and physiological activities at a resolution of individual organs under specific environments (Table 1) (Yan et al., 2004; Allen et al., 2005), originally derived from plant architectural models (De Reffye et al., 1988; Prusinkiewicz et al., 1988; Prusinkiewicz, 1998). For example, GreenLab (Hu et al., 2003), a typical FSPM, was initially tested for the key algorithms in biomass allocation (Song et al., 2003a) and morphological construction (Song et al., 2003b) for maize and was further developed with a systematic integration of interactive modules, i.e., developmental, biomass growth and partitioning, and architectural development and visualization (Yan et al., 2004; Guo et al., 2006); subsequently, the model was further generalized (Kang et al., 2008a) and widely applied to other crops (Dong et al., 2007; Kang et al., 2008b, 2012; Jullien et al., 2011). Simultaneously, many other FSPMs, model platforms, and tools have emerged worldwide (Fournier and Andrieu, 1999; Prusinkiewicz et al., 2000; Allen et al., 2005; Evers et al., 2006; Kniemeyer and Kurth, 2008; Pradal et al., 2008) with a particular focus on plant architectural development for diverse crops. In essence, FSPM has a robust physiological × architectural interaction at organ level in response to various environments (Hanan, 1997; Yan et al., 2004; Vos et al., 2010; El-Sharkawy, 2011; Henke et al., 2016; Postma et al., 2017; Schnepf et al., 2018; Zhou et al., 2020).

**Table 1:** The list of functional–structural plant models/platforms, brief description of characteristics, and basic functions.

**Model/platform**	**Brief description**	**Model basic functions**	**Model properties**	**References**
GreenLab	A model framework to integrate plant architecture and physiological function as growth cycles; each cycle composed of biomass production computation, biomass allocation, morphological construction; and applied to many crops.	Use mathematical equations and biological rules to simulate plant structural development and growth, and biomass partitioning among plant compartments, to mimic plant morphogenesis and its plasticity in response to various environments, allowing scaling down or up. Applied to different crops and plants.	3D development mainly on shoots; temporal scale as growth cycle with days depending on time to complete a metamer development; programmed with C++, Matlab, Java, Scilab	Hu et al., 2003; Song et al., 2003a,b; Yan et al., 2004; Kang et al., 2008a,b
L-Studio	A software system including a L-system-based simulation core program cpfg, and 3D plant modeling environments, and many models are developed in the L-studio platform.	Simulating plant growth and development and visualizing plant architecture according to specific tasks	2D or 3D platform; Time scale depending on specific application; programmed with L-system	Prusinkiewicz et al., 2000; Karwowski and Prusinkiewicz, 2004
GroIMP	An open-source modeling platform and the rule-based programming language XL (eXtended L-system), for realistic plants and conditions	Simulating plant architecture and physiological functions, and visualizing plant architecture in general.	3D; Time scale depending on specific application; programmed with Java-based XL	Kniemeyer and Kurth, 2008; Henke et al., 2016
OpenAlea	A user-friendly software platform for modelers to build models using a visual programming interface and provides a set of tools and models for plant modeling	Provide a visual and interactive interface to the inner structure of an FSPM specific application	3D; Time scale depending on specific application; programmed with Python	Pradal et al., 2008
GRAAL; GRAAL-CN	Plant organs (roots and shoots) development, resource acquisition (Carbon and Nitrogen) and management among organs, dynamic of imbalances between C-N metabolite	Analyse of the dynamic between morphogenetic process and assimilates (C-N) acquisition process during the vegetative development of individual plants	Schematic 2D; Daily scale; programmed with Java language	Drouet and Pages, 2003; Drouet and Pagès, 2007
NEMA	Nitrogen acquisition and distribution within aerial plant parts for wheat	Predict N content of each photosynthetic organs as regulated by Rubisco turnover which depends on intercepted light and a mobile N pool share to all organs	Schematic; Daily scale; programmed with L-system + C language	Bertheloot et al., 2011
L-Peach	A model developed using L-system formation. Plant structure development, carbon storage and remobilization	Use of L-system to simulate the development of plant architecture and explain the dynamically changing system of carbon accumulation and partition among organs	3D dynamic; Daily scale; programmed with L-system	Allen et al., 2005
EcoMeristem	Phenology, organ initiation as driven by meristem behavior, assimilate production (supply for carbon)	Simulate plant morphogenesis and phenotypic plasticity relying on adjustment methods relevant to C sink-sources variations	Schematic; Growth cycle as temporal scale; programmed with C language	Luquet et al., 2006
ADEL-Maize ADEL-Wheat	A model to drive plant development according to thermal time and simulate leaf architecture development using L-system.	Model maize and wheat 3D architectural development;	Shoot 3D dynamic; Daily scale; programmed with L-system	Fournier and Andrieu, 1999; Fournier et al., 2003
CN-Wheat	Carbon-nitrogen distribution in wheat plants (roots, shoots and grains)	Simulates the allocation of C-N into wheat culms in relations to photosynthesis, N uptake, metabolites turnover, root exudation and tissue death	Schematic; Process-based model Growth cycle as temporal scale; programmed with Python	Barillot et al., 2016
OpenSimRoot	An open-source modular infrastructure to simulate root architecture and function, with modules i.e., water uptake and xylem flow; tiller formation; evapotranspiration, etc.	Simulates root system architecture, the shoot, C, water and nutrient acquisition and utilization, root growth plasticity and geometric descriptors	Root 3D; Daily scale; programmed with C++	Postma et al., 2017
CPlantBox	A framework for simulating interaction between carbon and water flows; CPlantBox is an extension of the model CRootBox	Simulates the growth and development of a variety of plant architectures by combining with a mechanistic model of water and carbon flow	Schematic 2D; Hourly scale; programmed with C++, Python, R	Zhou et al., 2020
CRootBox	Root architectural development and root-soil interaction	Simulate dynamically and on field scale, based on computational science strategies, the responses of root architecture to environmental properties as well as the effects of roots on soil conditions	2D; Hourly scale; programmed with C++, Python, R	Schnepf et al., 2018

**Figure 1:**
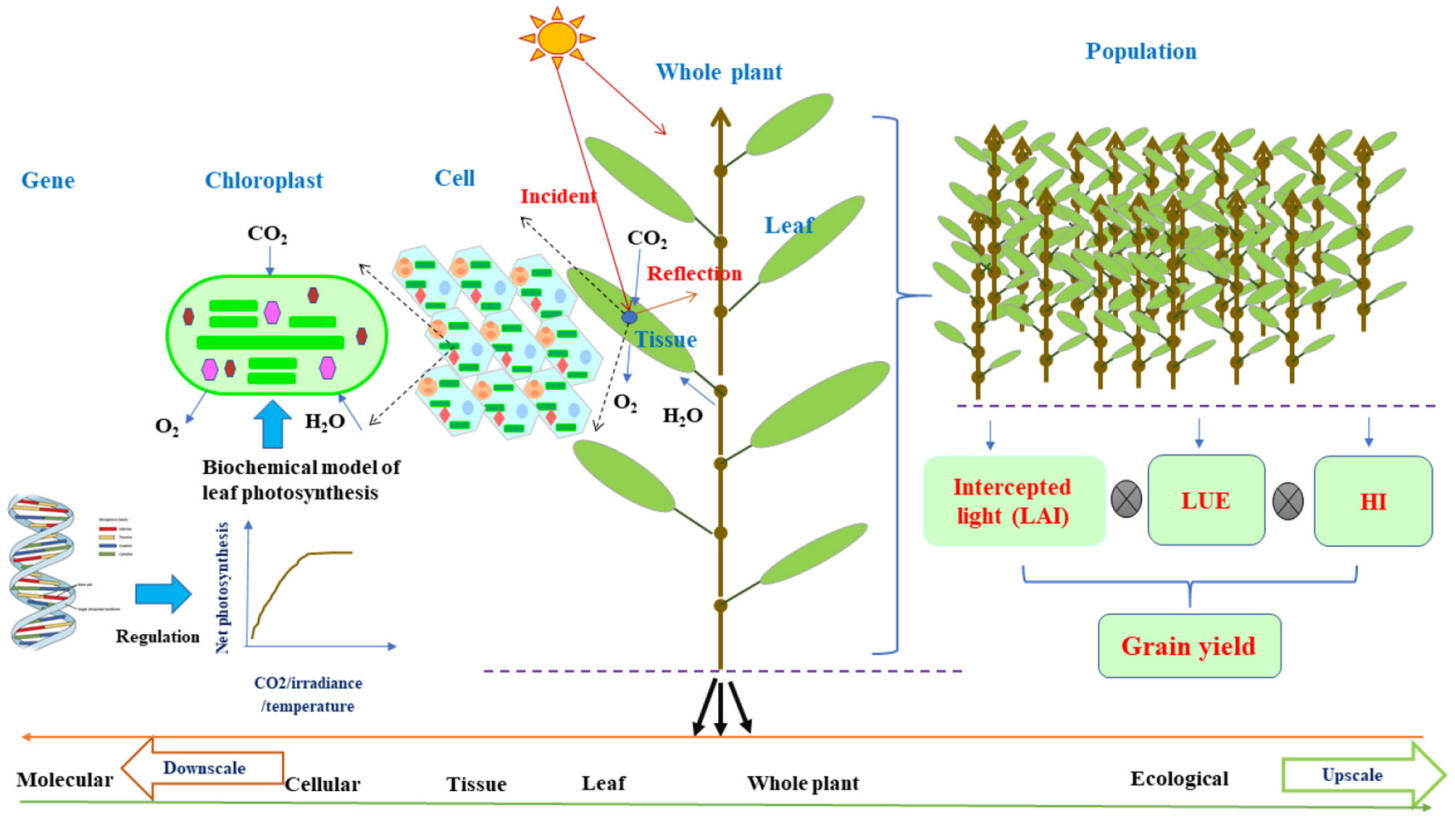
The schematic diagram of plant architecture and functional activities on the basis of individual organs for FSPMs downscaled to the cellular function or upscaled to the ecological function via leaf photosynthesis. Leaf photosynthesis can be decoded as a collection of cellular chloroplast photosynthesis per unit that can adopt leaf C_3_/C_4_ biochemical models (Farquhar et al., 1980) regulated by molecular network (Wu et al., 2016); while for field level, the estimation of grain yield from leaf photosynthesis is the intercepted light by the canopy as a function of LAI multiplying with LUE and HI. Leaf photosynthesis acts as a nexus in connecting cellular and molecular level to field level modeling. The curve shows net photosynthetic rate as a function of incident irradiance, CO_2_, H_2_O, and temperature. From left to right, the upscale from molecular to ecological level or vice versa for downscale from right to left. FSPMs, functional–structural plant models; LAI, leaf area index; LUE, light use efficiency; HI, harvest index.

It is noteworthy that FSPM framework is built on the multipurpose and multidisciplinary knowledge of structural and functional interactions on an organ level (Figure 1) and has been successfully applied for many plants under various environmental conditions, assisting in dealing with sustainable food production (Tardieu, 2003; Hanan and Prusinkiewicz, 2008; Evers et al., 2010; Vos et al., 2010; Guo et al., 2011). As a consequence, they have great potentials to attract more attention from scientists in various disciplines and can be the center of interest of debates in overcoming challenges arisen from the practice of crop production (Evers et al., 2018). Thus, in this study, we highlight the robust concepts of FSPMs with ecophysiological functions of a structural phytomer, flexible in allowing integration of disciplines for down- or upscaling. We then further illustrate unique potential roles of FSPMs in overcoming great challenges in sustaining crop productivity under environmental stresses. The following sections will demonstrate the role of FSPMs in: (i) assisting to design crop ideotypes with optimal use of resources, (ii) enhancing crop modeling ability by assisting to link phenotypes to genotypes, (iii) improving the efficiency and accuracy of molecular breeding, and (iv) guiding plant phenotyping for efficient breeding. Integration of favorable ideotype identification, traits discovery, and the reduction of the gap between phenotypes and genotypes collectively contribute to developing new cultivars for stable and sustainable production under adverse environments.

## Functional–Structural Plant Models Guide Plant Ideotype Design

A crop ideotype, originally defined by Donald (1968), is the combination of collective elite traits that may control crop growth and development, grain yield, and stress tolerance in specific environments (Qi et al., 2010; Andrivon et al., 2012; Rötter et al., 2015). Conventional breeding efforts depend on experienced breeders to combine the alleles in tedious and time-consuming field trials. However, the FSPM can provide *in silico* plants that aid to conduct virtual trials in achieving theoretical ideotypes by adjusting any combination of traits (Tardieu, 2003; Song et al., 2013; Picheny et al., 2017) and testifying them by rigorous field trials.

Functional–structural plant model requires a supply of resources including irradiance, nutrients, and H_2_O as a fuel and building materials for organ kinetics and morphophysiological activities as well as comprehending how to manipulate such resources in 3D development and ecophysiology of each phytomer precisely for optimal plant architecture (Ourry et al., 2001). Further, resource distribution within the plant confines many aspects of crop growth and development and grain yield formation as well as the risk of disease infection. Consequently, the crop performance is regulated considerably by the way crop interacts with the environments involved in the processes that lead to final product formation and quality.

One of prime features in FSPM is a plant composed of a network of structural units such as axes, internodes, leaf tips, and axillary buds (Vos et al., 2010; Sievänen et al., 2014). This offers the possibility to study and model the development and functioning of each metamer/organ and the interaction of each metamer via resource competition. Being a prerequisite factor for plant photosynthetic process, light absorption/interception is one of the important factors in determination of crop yield. The introduction of modeling paradigm that focuses on the spatial design of plant architectural traits and their development gives the opportunity to explore light absorption and photosynthesis for each structural element, biomass partitioning, and grain yield (Chelle and Andrieu, 1998; Cournède et al., 2008; Sarlikioti et al., 2011b; Da Silva et al., 2014; Sievänen et al., 2014; Christensen et al., 2018). In this context, Sarlikioti et al. (2011a) performed simulations with FSPM of tomato crop to define plant ideotype for optimal light distribution, absorption, and canopy photosynthesis. This study defined two ideotypes scenarios that exhibited an increase in light absorption, resultantly higher canopy photosynthesis, which, in turn, may potentially lead to an enhanced yield. Interestingly, they identified that internode length and leaf shape are the most essential architectural traits to be manipulated in optimizing light absorption. This demonstrates that plant architectural information may have significant importance in modern breeding to design genotypes with respect to efficient light absorption and canopy photosynthesis (Sarlikioti et al., 2011b).

Furthermore, the importance and implication of plant architecture in the identification of plant ideotypes with respect to light partitioning capacity in a crop mixture is highlighted (Barillot et al., 2014). In fact, in this study, the authors developed a deterministic model of pea (L-pea) with modules for vegetative topological development and organ dynamics, linked it with Architectural Model of Development Based on L-systems (ADEL)-Wheat (Fournier et al., 2003) in a common L-system platform, and applied the model to a cropping system of pea and wheat to assess light partitioning. Results illustrated that quantitative variation of architectural traits is a determining factor for light partitioning (Barillot et al., 2019) and that in intercropping systems (such as wheat and pea), light capture is principally linked to architectural characteristics. Such findings can facilitate the design of crop genotypes adapted to intercropping by capturing morphological traits that can be incorporated into modern breeding programs (Louarn et al., 2020). These studies demonstrated how far beyond can FSPMs provide a finer insight of light absorption and partitioning within plant canopy and also deliver tools that help to establish a fine set of architectural traits for maximizing canopy photosynthesis, allocation of assimilates to growing organs, and ultimately crop yield (Sarlikioti et al., 2011b; Teichmann and Muhr, 2015). Apart from abiotic environments, pea architectural details are reported to affect spatiotemporal epidemic development for *Ascochyta* blight (Le et al., 2009) and an ideotype with the combinations of optimal architectural traits is shown to minimize the epidemic development of pests and diseases in crops (Andrivon et al., 2012).

In addition to shoot ideotypes, root ideotypes have been explored in maximizing the uptake of resources in the soil. A “steep, cheap, and deep” ideotype with the ability of optimizing the acquisition of water and nitrogen was proposed by Lynch (2013). An ideotype of root system for efficient nitrogen acquisition in intensive cropping system was proposed by Mi et al. (2010) and further updated with more detailed root architecture including root branching, angle, and distribution (Mi et al., 2016). A novel irrigated ideotype with high resource use efficiency was proposed by Schmidt and Gaudin (2017). The functional–structural plant modeling has been applied to identify the ideotype of root system drought resistance for breeding (Ndour et al., 2017).

## Functional–Structural Plant Models Assist in Molecular Design Breeding

The modern molecular breeding (Moose and Mumm, 2008) with the guidance of crop design by employing the knowledge and tools arisen from contemporary functional genomics is fairly effective for breeding new cultivars (Hammer et al., 2016). A considerable literature has been dedicated to understanding how crop/plant modeling could help to decode complex traits for guiding molecular breeding (Hammer et al., 2005; Yin et al., 2005; Chapman, 2008). It is an extended form of the standard breeding approach by the prediction of genotypic breeding. As such, it allows the breeding procedure to be simulated and optimized prior to being tested in the field, thus increasing breeding efficiency and predictability (Hammer et al., 2006, 2016; Wan, 2006). Designing superior crop cultivars would be affordable for breeders due to genetic basis of agronomically important traits and allelic variations at those loci made available (Wan, 2006; Wang et al., 2011).

The framework and concept of FSPMs to represent the plant as a network of elementary units, i.e., phytomers and their structural–functional feedback, provide great opportunities to comprehend plant biological organization from molecular level to whole plant (Figure 1) (Hanan and Prusinkiewicz, 2008). There are possibilities to connect the whole plant trait to fundamental biology via FSPMs in accordance with the behavior of the entire plant systems biology (Letort et al., 2008; Xu and Buck-Sorlin, 2016). Molecular design has been attracting great interest in plant breeding programs (Wang et al., 2011). The link up of a given model measurable trait and tangible quantitative trait loci (QTL) is the key fact that makes crop models or FSPMs an integral tool for crop molecular genetics research and breeding (Tardieu et al., 2005; Quilot et al., 2006; Letort et al., 2008; Semenov and Halford, 2009). Accordingly, Xu et al. (2011) developed a model system of rice that represents plant structural kinetics in combination with ecophysiological processes using FSPMs and interactive modeling platform Growth Grammar-related Interactive Modelling Platform (GroIMP) (Kniemeyer and Kurth, 2008) along with the graph-based relational growth grammar formalism (Kurth et al., 2004), which is an extended L-system formalism. This prototype constitutes the first effort of a model system of rice FSPMs that will prominently integrate information on QTLs, environments, and their interactions in a network. This could help further for designing molecular specific traits in crop systems biology or in breeding. Plant under water stress has different responses underlined by various physiological processes that could account for emergent behavior. Associating alleles with particular responses will help to identify alleles for maintaining growth under stress (Tardieu et al., 2005). Leaf elongation rate depends on environmental variables, e.g., temperature, evaporative demand, and soil water status, so QTLs for these variables were established, enabling to predict the responses to different climatic conditions. The identification of QTLs in this study offers opportunities for improving drought adoption mechanisms via molecular breeding to design and assess traits that were elusive in previous selection study.

Overall, as a mechanistic and comprehensive tool, the FSPM can be used in molecular breeding work to assist in the design of new plant prototype. They will be for sure embrace the system design in addition to the synthesis of data and prediction of quantitative behavior, as proposed by Yin and Struik (2008) for future modeling of crop systems biology.

## Functional–Structural Plant Models Enrich Architectural Details for Crop Models

Crop models are usually capable in predicting crop phenology, biomass, and grain yield under various soil and climate conditions including abiotic stresses (Jones and Kiniry, 1986; Sinclair and Seligman, 1996; Wang et al., 2019). Initially, such models have been employed to assist crop management in a farming system with a simplification of plant architectural details. The model design based on robust physiological principles is in accordance with the systems biology (Hammer et al., 2005; Yin and Struik, 2009), e.g., Genotype-by-Environment Interaction on Crop Growth Simulator (GECROS) (Yin and van Laar, 2005). Crop models are shown to be promising to connect with molecular level mechanisms in assisting plant breeding for complex traits, e.g., drought tolerance (Hammer et al., 2005; Chapman, 2008). For example, a linkage between crop models and leaf biochemistry models has been proposed to reflect the adjustment of biochemical reaction in grain yield for crop improvement (Wu et al., 2016; Yin et al., 2018). Often, leaf area index is required in driving crop photosynthesis and productivity in modern crop models such as Decision Support System for Agrotechnology Transfer (DSSAT) (Jones et al., 2003) and Agricultural Production Systems Simulator (APSIM) (Hammer et al., 2010). Detailed root architecture is essential in investigating water or nutrient absorption under abiotic stresses (Fang et al., 2009; Hammer et al., 2009). Thus, the resolution of representing plants at organ level is helpful to enhance the capacity of crop modeling for the precise description of a plant.

Functional–structural plant models have been developed with a particular focus on a delicate description of plant structure, initially known as plant architectural models or virtual plants (De Reffye et al., 1988; Barthelemy and Caraglio, 2007). FSPMs add a structural dimension to conventional crop models (Vos et al., 2007). Upon the advent of FSPMs, supply fine details of plant architecture is reinforced for likely use in crop models (Wernecke et al., 2000; Vos et al., 2007; Fourcaud et al., 2008; Feng et al., 2014). For example, the GreenLab model (Hu et al., 2003; Yan et al., 2004; Kang et al., 2008a) takes fundamental ecophysiology in calculating biomass production and partitioning and links physiology with architectural models (Slavíková, 1980) for more precise prediction of crop production (De Reffye et al., 2009). Further, this model has been calibrated for many plants and has been shown to be able to accurately reproduce plant growth and architecture with phenotypic plasticity (Dingkuhn et al., 2005).

In earlier days even when crop models reached maturity (Sinclair and Seligman, 1996), high resolution of canopy architecture is not necessary and time-consuming in farming-scale simulations. Given fundamental biological functions closely associated with plant architecture, the description of shoot and root architecture may be valuable in crop models. Thus, it deserves more attention to improve the resolution of canopy architectural details, allowing matching the heterogeneity of environmental resources required for precision computation of crop productivity and design in molecular breeding with crop models (Dai et al., 2004; Evers et al., 2010; Yin et al., 2018).

## Functional–Structural Plant Models Assist in Plant Phenotyping

Assessment of qualitative and quantitative traits rapidly, known as plant phenotyping (Granier and Devis, 2014), helps to explore functional diversities or performances of different plants in given environmental conditions. Plant phenotyping calls for strategies that include collecting data from the experiment and submitting those data to a crop/plant model that will provide predicted crop traits (Messina et al., 2015). The models should be, therefore, able to simulate G × E interactions and the resulting phenotypic plasticity (Dingkuhn et al., 2005). Within the context, Luquet et al. (2006) developed EcoMeristem, a FSPM explicitly conceived to simulate rice crop phenotypic plasticity on the basis of meristem behavior and associated adjustment processes (Dingkuhn et al., 2005). Luquet et al. (2012a) illustrated the practicability of the model while using it to explore the phenotypic and genetic diversity of early vigor and drought regulation in rice. In their study, according to optimized parameters, the model accurately simulated plant leaf area, plant height, and shoot dry weight under both the well-watered and drought conditions. This shows the capacity of the model to reproduce the behavior of morphophysiological traits. Model parameters can provide phenotypic data less noised by “genotype by environment interaction,” as the parameters showed less replicate effect compared to corresponding measurements (Rebolledo et al., 2012). In this context, a theoretical study based on linking parameter values of GreenLab (Yan et al., 2004; Guo et al., 2006) to hypothetical genes was done by Letort et al. (2008). This study simulated the virtual phenotypes resulting from hybridization of homozygous parents, showing that this virtual phenotype resulted from population cross could be used in QTL identification for further breeding use. Therefore, for building new ideotype concepts of phenotyping, FSPM approaches are needed because they help to integrate knowledge of physiology, explicit organ 3D characteristics, and genetics via such a bridge between plant science and functional genomics.

## Functional–Structural Plant Models Amenable to Cross Disciplines and Scales

Functional–structural plant models are equipped with interactive modules built for precisely exploring plant morphogenesis, development, and growth in the context of environmental cues. They are specifically valuable in synthesizing research understanding and integrating discipline knowledge to generate tools with descriptive and mechanistic potentials (DeJong et al., 2011; Sievänen et al., 2014; Louarn and Song, 2020). Owing to their multidisciplinary characteristics, FSPMs are based on concepts, tools, and frameworks that emanate from various disciplines, thus the development of FSPMs involves scientists with a wide range of backgrounds including crop physiologists, plant biologists, plant ecologists, computer scientists, and agronomists, etc (Figure 1).

A modeling study with FSPMs may generate massive data at different scales, thus managing these data constitutes a new challenge for modelers (DeJong et al., 2011). Therefore, the integration of data acquisition techniques involving laser scanning, confocal laser imaging, and X-rays, underlined by remote sensing approach, led to the design of “3D” FSPMs that work at various spatial-temporal scales. Based on laser scanning technology, Boudon et al. (2014) developed algorithms for automatic identification of plant elementary units, further used to parameterize FSPMs and evaluate them, according to accurate and real generated data. Hakala et al. (2012) showed that the feasibility of analyzing spectral characteristics of the Light Detection and Ranging (LiDAR) 3D point clouds generates future prospect in FSPMs for identification of plant parts and their physiological conditions. The development of FSPMs provides platforms for computational modeling that depends on appropriate software and programming languages (Sievänen et al., 2014). For example, as established concepts are frequently adjusted to provide new approaches in modeling studies, L-system was an open tool and concept (Prusinkiewicz and Lindenmayer, 1990) with a multi-modules system for integrating previous modeled aspects of carbon dynamics (Allen et al., 2005), apical dominance (Prusinkiewicz et al., 2009), and biomechanics (Taylor-Hell, 2005; Prusinkiewicz et al., 2007) into a well-structured FSPM (Cieslak et al., 2011). This is illustrated in the study of Ong et al. (2014), where the programming language XL and GroIMP platform have been used to explore models of plant growth that allow appropriately the use of several structural scales in plant description, highlighting the multi-scalar potentials of FSPMs. The result pointed out 3 contrasting models that show the way for combining information from various scales in the models. These are top-down, bottom-up, and within a range of scales from microscopic cell-level process to macroscopic level of plants. The integration of discipline knowledge, techniques, and concepts for the development of FSPMs and explicit tools for usage beyond the individual discipline could produce user-oriented multifaceted models for application in studying complex systems (Boote et al., 1996; Sievänen et al., 2014).

## Functional–Structural Plant Models Solve Challenges in Crop Production

Functional–structural plant models have been deployed to comprehend morphological, physiological, and biological processes that drive development, growth, and yield formation of crops in various environmental conditions and to simulate the consequences of crops × environments including the effect of biotic and abiotic stresses (Hanan and Prusinkiewicz, 2008; Sievänen et al., 2014). FSPMs offer considerable potentials for tackling current challenges including food security for greater human population and sustainability in the context of biotic/abiotic stress due to climate change (Chapman, 2008; Wang et al., 2019). Inherently, one of the greatest bottlenecks in crop production is managing biotic and abiotic factors that significantly reduce crop production (Maiti and Pratik, 2014). The usefulness of FSPMs at tackling these issues has been demonstrated (Garin et al., 2014; Gigot et al., 2014). For example, the drought stress occurring at crop establishment stage has been deleterious to rice crops (Courtois et al., 2000). The only way to alleviate that is for the plant to acquire sufficient resources and avoid soil evaporation and weed rivalry (Zhao et al., 2006). This is termed as “early vigor,” which confers drought avoidance ability in rice crops (Zhang et al., 2005). As the FSPM allows formalizing integratively, the genetic (G) × environment (E) bases of elemental process-based traits and their linkages, it was able to simulate genetic diversity of rice early vigor and its drought regulation (Luquet et al., 2012b). FSPM concepts were applied in the EcoMeristem (Luquet et al., 2006) to investigate the existence of negative linkages between the capacity of proper plant establishment and its drought tolerance (Luquet et al., 2012b). Indeed, those identified negative linkages could be attributed to the variation of resources *per se* and also the reaction of sink activities to available resource. The result of this study would eventually help rice breeders to better co-select early vigor and drought tolerance traits (Luquet et al., 2012a).

In a former study, a modeling framework was produced to simulate foliar fungal epidemics based on the OpenAlea platform (Pradal et al., 2008). This study is designed by implementation of two different pathosystems and yielded the simulation of the effect of canopy structural traits on fungal dissipation. This paves the way for modeling the complex dynamics of crop pathosystems for a good understanding of interactions that will probably make better protective strategies (Garin et al., 2014). The study by Gigot et al. (2014) proposed as a strategy for managing splash-dispersed fungal pathogen in wheat to define cultivar (whether sensible or tolerant) proportion as a function of host resistance capability. FSPM technique used in this study referred to a virtual 3D plant model, integrated to a module that predicts splash droplet dispersion of the fungal pathogen and the host resistance in wheat. This highlights how FSPMs, through its spatial-temporal characters, can make itself useful for understanding issues related to the dissipation of disease within plants.

## Challenges of FSPM Development

After more than 2 decades of evolution of FSPM (Vos et al., 2010; Louarn and Song, 2020; De Reffye et al., 2021), the model has become widely known due to the continuous efforts from the pioneers in both the plant architectural modeling and functional–structural plant modeling community (Prusinkiewicz et al., 1988; Hanan, 1997; Hu et al., 2003; Godin and Sinoquet, 2005). The models have achieved great success in algorithms and prototypes for different plants or crops under various environments, receiving more attention nowadays and in future (Louarn and Song, 2020). The above paragraphs also demonstrated the great capacity for FSPMs in addressing the challenges in crop science. Despite this, we have identified constraints in both the modelization and practical aspects that may limit the potentials of extensive applications for FSPMs.

First of all, it is known that the major strength in FSPMs is the fine simulation of explicit plant morphogenesis, 3D architecture, and architectural development; nevertheless, the key functional parts in many FSPMs are, to some extent, based on or adopted from the physiological processes used in conventional crop models, in particular, in the beginning when to illustrate the role of FSPMs by integrating both the plant architecture and physiological functions (De Reffye et al., 2009), which is still widely used. For example, the estimation of canopy photosynthesis in many FSPMs is based on empirical light extinction within the canopy as a function of leaf area index (Vos et al., 2010; Pao et al., 2021) rather than mechanistic interaction of irradiance with individual leaves, though there are many studies available in investigating such interaction (Buck-Sorlin et al., 2011; Sarlikioti et al., 2011b). In addition, modeling transfer of incident light energy to the chemical energy in the form of carbohydrate in leaves can be realized by mechanistically biochemical model of leaf photosynthesis (Farquhar et al., 1980; Wu et al., 2016). Hence, the mechanistic process of carbohydrate should be introduced in the novel model stage. Taken another example, there is attempt in mathematically simulating biomass allocation among individual growing organs (Kang et al., 2008b; Reyes et al., 2019). However, the biomass flow into the sink governed by fundamental cellular activities is rarely studied. As FSPMs are maturing, it is time and necessary to mimic the mechanistic, physiological process rooted from an organ activity, which will be desirable and boosted in future development. It needs to dismantle the integrative sink strength into fundamental cellular activities driven by sucrose unloading and following sucrose degraded into glucose and fructose, which is regulated by a series of enzymes and genes (Ruan, 2014). Consequently, the participation from crop/plant scientists and a closer collaboration between model developers and those field scientists should be more encouraged for model development and practice, though the models have been initially developed by joint efforts from mathematicians, modelers, and computer scientists as well as with the participation of agroforestry scientists.

Practically, as the model considers both the fundamental biological processes and plant architecture, even visualization, it will require substantial computation time. We got to admit that the computation power has made great progress over the last decades. Nonetheless, it is still a major concern for the models applied to the complicated system in practice by integrating details including soil and atmosphere environments and crops. To address this, for instance, the visualization is made in separate rendering program depending on if it is required, e.g., GreenLab (Kang et al., 2008a,b). In addition, to facilitate the application, it is essential to have a user-friendly interface and practice the software or tools without knowing much about underlying model algorithms. At the current stage, the use of models is not easy for users in crop science who are not with fairly good backgrounds of FSPMs. It takes a while to train new users about how to use tools and software.

## Conclusion and Future Insights

Crop science is confronted with the challenge for substantial improvement of crop productivity under climate change for increased human population. This requires elite cultivars tolerant to adverse environments to be bred. The ideotypes, traits, phenotypes, and molecular design breeding were integrated in a system via FSPMs for more efficient breeding. FSPM, by tracing organ kinetics, microenvironments, and their interactions, enables to understand and explore how the complex crop system work, which allows the model to be downscaled to the molecular level or upscaled to the plant community in a faithful way to the systems biology. Also, FSPMs may be envisaged to generate more substantial details arisen from the analysis of genotypic and environmental interaction at different scales. FSPMs provide algorithms, platforms, and tools in advancing the frontiers in crop science from molecular design to phenotypic-guided breeding, by which, sustainable crop production under adverse environments may be achieved. On the other hand, the existing and promising applications in advancing crop science will result in the evolution of FSPMs. Though the attempt for FSPMs applied to advancing the frontiers in crop science has been demonstrated, there is still much more study to be done in fulfilling the potentials. This includes bringing together scientists in different disciplines to work closer than ever in guiding molecular design for precise breeding via FSPMs.

## Author Contributions

SS drafted the manuscript. ZW and WS helped in manuscript draft. PR advised the manuscript. BC improved the writing. GL and YS conceived the idea and finalized the manuscript. All authors contributed to the article and approved the submitted version.

## Funding

The study was financially supported by the National Key R&D Program of China (Grant nos. 2017YFD0301307 and 2017YFD0300204-3).

## Conflict of Interest

The authors declare that the research was conducted in the absence of any commercial or financial relationships that could be construed as a potential conflict of interest.

## Publisher's Note

All claims expressed in this article are solely those of the authors and do not necessarily represent those of their affiliated organizations, or those of the publisher, the editors and the reviewers. Any product that may be evaluated in this article, or claim that may be made by its manufacturer, is not guaranteed or endorsed by the publisher.
